# Effect of weight loss interventions on metabolomic signatures in obese children with insulin resistance

**DOI:** 10.1007/s00726-024-03409-2

**Published:** 2024-08-30

**Authors:** Xiaoguang Liu, Lin Zhu, Jingxin Liu, Zichen Nie, Wenjun Qiu

**Affiliations:** 1 School of Sport and Health, Guangzhou Sport University, Guangzhou, China; 2 Guangdong Provincial Key Laboratory of Physical Activity and Health Promotion, Guangzhou Sport University, Guangzhou, China; 3 Physical education and sports school, Soochow University, Suzhou, China; 4 Harbin Institute of Technology, Shenzhen, China; 5 Zhongkai University of Agriculture and Engineering, Guangzhou, China

**Keywords:** Obesity, Children, Insulin resistance, Metabolites

## Abstract

The obesity epidemic among children has become a major public health issue, and the presence of childhood insulin resistance (IR) has been demonstrated prior to the onset of type 2 diabetes mellitus. However, it is unclear whether the metabolomic signature is associated with weight loss interventions in obese children with IR. Thirty-six obese children with IR were selected from the weight loss camp (Shenzhen Sunshine Xing Yada health Technology Co., LTD). Clinical parameters were collected before and after weight loss intervention. Targeted metabolomics of plasma samples was performed by ultra-performance liquid chromatography coupled to the tandem mass spectrometry, and principal component analysis, variable importance in projection, and orthogonal partial least squares discriminant analysis were used to obtain the differentially expressed metabolites. Pathway analysis was conducted with the Homo sapiens (HSA) sets in the Kyoto Encyclopedia of Genes and Genomes. We used machine learning algorithms to obtain the potential biomarkers and Spearman correlation analysis to clarify the association between potential biomarkers and clinical parameters. We found that clinical parameters and metabolite clusters were significantly changed in obese children with IR before and after weight loss intervention. Mechanistically, weight loss intervention significantly changed 61 metabolites in obese children with IR. Furthermore, 12 pathways were significantly changed. Moreover, the machine learning algorithm found 6 important potential biomarkers. In addition, these potential biomarkers were strongly associated with major clinical parameters. These data indicate different metabolomic profiles in obese children with IR after weight loss intervention, providing insights into the clinical parameters and metabolite mechanisms involved in weight loss programs.

**Supplementary Information:**

The online version contains supplementary material available at 10.1007/s00726-024-03409-2.

## Introduction

The obesity epidemic among children and adolescents continues to worsen and remains a major public health issue in China (Pan et al. 2021) and around the world (Lister et al. 2023). Obesity in childhood correlates with a higher likelihood of encountering cardiovascular issues, like hypertension, ongoing inflammation, and insulin resistance. These factors can potentially lead to the progression of diabetes and heart diseases as one grows into adulthood (Bleich et al. 2018; McPhee et al. 2020).

IR is characterized as a state of decreased responsiveness of insulin-targeting tissues to high physiological insulin levels and is considered the pathogenic driver of numerous cardiovascular diseases, nonalcoholic fatty liver disease (NAFLD), and type 2 diabetes mellitus (T2DM) (Lee et al. 2022). T2DM can cause chronic kidney disease, retinopathy, lower limb amputation, myocardial infarction, stroke and other complications. IR observed in the pediatric population is a significant concern due to the established link between the early manifestation of this metabolic condition and a heightened susceptibility to microvascular complications (Huang et al. 2022).

The best approach to the prevention and treatment of IR and T2DM is lifestyle alteration. Numerous studies suggest that exercise can effectively reduce IR in obese children(Liu et al. 2020)^,^ (Elisa et al. 2016). Aerobic exercise could decrease the level of circulating branch-chain amino acids (BCAAs), which is the best metabolic predictor of improvement in insulin sensitivity in overweight humans (Glynn et al. 2015). Moreover, emerging studies have demonstrated that the plasma metabolome profile changes with resistance training and endurance training, revealing the exercise-induced changes in metabolites that may help explain the underlying mechanisms of physical exercise on metabolic health (Mardinoglu et al. 2018)^,^ (Grapov et al. 2019)^,^ (Short et al. 2019). In addition, in our previous study we found that weight loss intervention improved cardiometabolic health in children with metabolic syndrome (Liu et al. 2021). However, few studies have explored the effects of weight loss interventions on metabolites in obese children with IR. Thus, the aim of this study is to determine the metabolite changes in obese children with IR and to screen potential biomarkers for IR obesity phenotypes. On this basis, we explored the effects of weight loss intervention on the metabolic profile and the potential metabolic mechanism in obese children with IR.

## Materials and methods

### Experimental design and participant inclusion criteria

The research was designed as a comparative quasi-experiment. Children with obesity were defined as having a body mass index (BMI) that corresponded to the Chinese criteria (WS/T 586—2018). This standard was drafted by Peking University Institute of Child and Adolescent Health, China Center for Disease Control and Prevention, Institute of Nutrition and Health, Maternal and Child Health Center of the Chinese Center for Disease Control and Prevention (Supplementary Table 1). Obese participants (18 boys and 18 girls) aged 10–13 years were selected from the weight loss camp (Shenzhen, China) from June to September 2019. All participants and their parents signed informed consent forms. They can discontinue from this camp if they want. Besides, this study was approved by the Ethical Committee of the Guangzhou Sport University (No. 2018LCLL-008).

Basic clinical data were collected from all obese children, including height, body weight, gender, age and comorbidities (heart disease, kidney disease and hepatitis B, chronic illnesses). All participants were diagnosed without any of the above diseases and had no history of medication. There were thirty-six obese children diagnosed with IR. Obese children with a homeostasis model assessment insulin resistance (HOMA-IR) threshold of > 2.6 were identified in the IR group(Feroe et al. 2016)^,^(Burrows et al. 2015). HOMA-IR was calculated using the following formula: $\:HOMA-IR=\frac{FINs(\mu\:U/L)\ast\:FPG(mmol/L)}{22.5}$ (Liu et al. 2021).

During the 30-day extreme weight loss exercise intervention in weight loss camp, all subjects received the standard exercise combined with the diet control program. All subjects participated in two training sessions per day. Each daily training session began with a 30-minute general warm-up, followed by 80 min of exercise training at a maximal heart rate (HRmax) of 50-80%, and concluded with 10 min of relaxation. Dietary control for each group was designed based on the resting metabolic rates (RMR). Before the intervention, each participant’s RMR was measured by indirect calorimetry (Supplementary Table 2). The details of the intervention were described in a previous study (Liu et al. 2021).

**Fig. 1:**
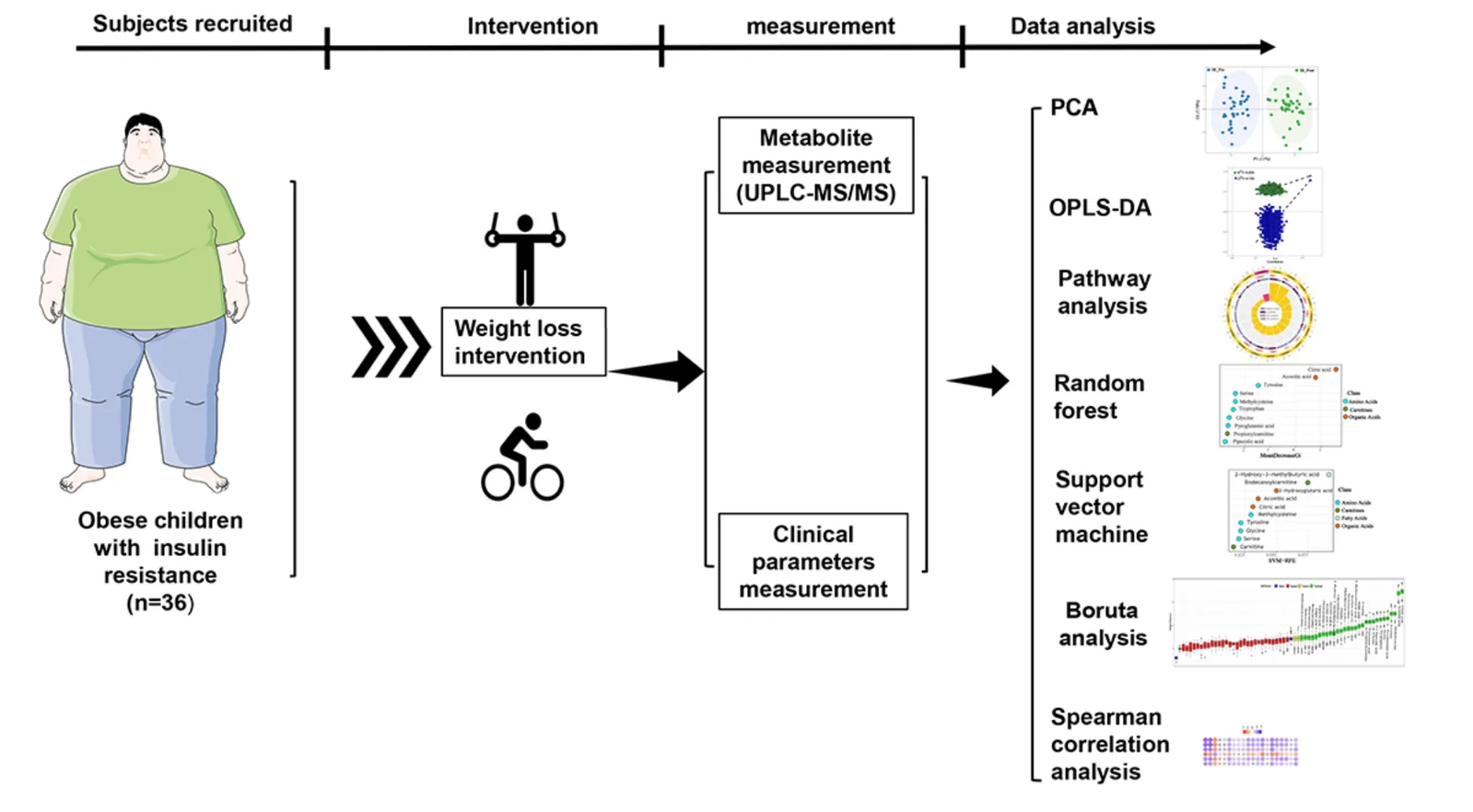
Flowchart of the study analyses in this work

To investigate the metabolomics characteristics of obese children with IR, we profiled plasma samples from the IR groups before and after weight loss intervention program (Fig. 1).

### Clinical parameter measurements

Before eating in the morning, every participant’s height, weight, chest circumference (Chest C), waist circumference (WC), hip circumference (Hip C), waist to hip rate (WHR), thigh circumference (Thigh C) and calf circumference (Calf C) were measured. We used the body composition analyzer (T-SCAN PLUS, Korea) to measure body composition, including fat-free mass (FFM), skeletal muscle mass (SMM), body fat percentage (BFP), and fat mass (FM).

Systolic blood pressure (SBP) and diastolic blood pressure (DBP) were measured by an electronic blood pressure monitor (OMRON HEM-1020, China). The levels of triglycerides (TGs), total cholesterol (TC), high-density lipoprotein cholesterol (HDL-C), and low-density lipoprotein cholesterol (LDL-C) in blood were analyzed by enzyme assays. Fasting plasma glucose (FPG) levels were determined using the glucose oxidase method (YeaSen, Cat: 60408ES60), while fasting insulin (FINS) levels were assessed through an enzyme-linked immunosorbent assay (Beyotime, Cat: PI608).

### Targeted metabolomics profiling

The fasting plasma samples were collected via the antecubital vein with heparin sodium as an anticoagulant at the end of a 30-day extreme weight loss exercise program.

The plasma samples were allowed to stand for 30 min before being centrifuged at 4 °C for 10 min at 3000 g. Following centrifugation, the samples were rapidly frozen in liquid nitrogen and subsequently stored at − 80 °C. The targeted metabolomics of plasma samples was performed by Metabo-Profile (Shanghai, China). The sample preparation procedures were performed according to previously published methods with minor modifications (Xie et al. 2021). Briefly, 20 µL of plasma was mixed with 120 µL of methanol containing an internal standard (Supplementary Table 3) using a vortex, then centrifuged to extract the metabolites. All internal standards were commercially purchased as previous published methods.

Thirty microliters of supernatant was subjected to derivatization with 3-nitrophenylhydrazine (3-NPH) and N-(3-(dimethylamino)propyl)-N′-ethylcarbodiimide (EDC)·HCl (Sigma‒Aldrich, St. Louis, MO, USA). Subsequently, the derivatized samples were analyzed by ultra-performance liquid chromatography coupled to tandem mass spectrometry (UPLC‒MS/MS) (ACQUITY UPLC-Xevo TQ-S, Waters Corp., Milford, MA, USA). All of the standards were obtained from Sigma‒Aldrich (St. Louis, MO, USA), Steraloids Inc. (Newport, RI, USA) and TRC Chemicals (Toronto, ON, Canada). The raw data files generated by UPLC‒MS/MS were processed using Targeted Metabolome Batch Quantification (TMBQ) software (v1.0, HMI, Shenzhen, Guangdong, China) to perform peak integration, calibration, and quantitation for each metabolite. The self-developed platform iMAP (v1.0, Metabo-Profile, Shanghai, China) was used for statistical analysis.

### Quantification and statistical analysis

We used Shanghai Metabo-Profile (self-developed platform iMAP v1.0) for data analysis. Principal component analysis (PCA) and orthogonal partial least squares discriminant analysis (OPLS-DA) were also performed. Variable importance in projection (VIP) was obtained based on the OPLS-DA model. Differentially expressed metabolites (DEMs) were regarded as statistically significant when the VIP of the metabolite was > 1 and the *p* value was < 0.05.

Finally, pathway analysis was conducted with the Homo sapiens (HSA) sets by the Kyoto Encyclopedia of Genes and Genomes (KEGG). Support vector machine (SVM), random forest (RF), and logistic regression were used to establish a diagnostic model. Statistical algorithms were adapted from R studio’s widely used statistical analysis software packages (http://cran.r-project.org/).

## Results

### Effects of weight loss on the clinical characteristics of obese children

**Fig. 2:**
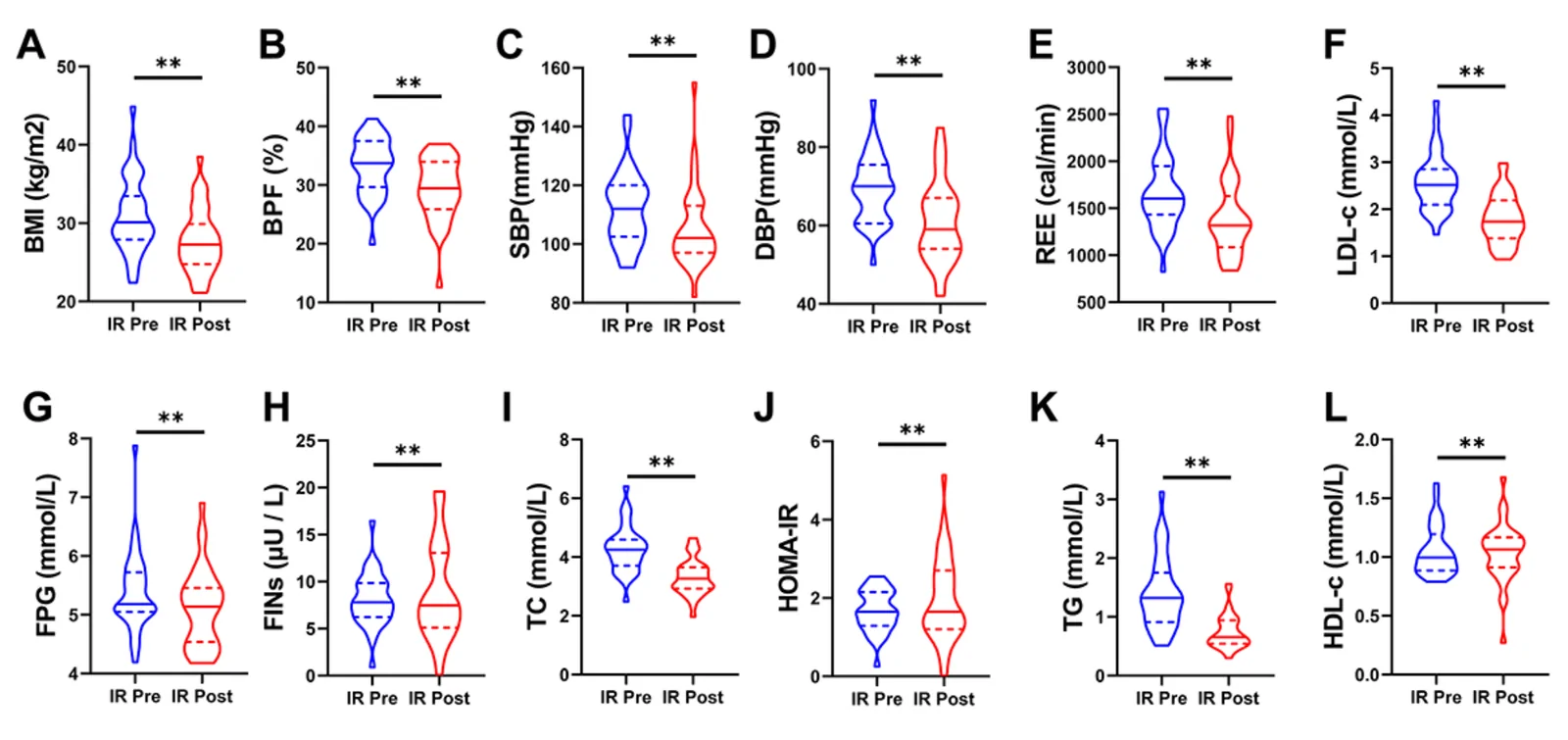
The Changes of cardiometabolic risk factors in IR group before and after weight loss intervention. *p < 0.05 between corresponding groups. **p < 0.01 between corresponding groups

Multiple previous studies have shown that lifestyle modification can effectively improve physical condition. In this study, obese children with or without IR participated in 4-week lifestyle modification interventions. Body mass index (BMI), systolic blood pressure (SBP), diastolic blood pressure (DBP), resting energy expenditure (REE), low-density lipoprotein cholesterol (LDL-C), high-density lipoprotein cholesterol (HDL-C), fasting blood glucose (FPG), fasting insulin (FINS), total cholesterol (TC), Triglycerides (TGs), homeostasis model assessment of insulin resistance (HOMA-IR) and HOMA insulin sensitivity (HOMA-IS), body weight (BW), chest circumference (Chest C), waist circumference (WC), hip circumference (Hip C), waist to hip ratio (WHR), thigh circumference (Thigh C), calf circumference (Calf C), fat mass (FM), fat-free mass (FFM), skeletal muscle mass (SMM), body fat percentage (BFP) were significantly decreased in children with IR after lifestyle modification (Fig. 2) (Table 1). No difference was observed in resting O_2_ and resting CO_2_ between before and after weight loss intervention (Table 1).

**Table 1:** Clinical characteristics of obesity IR children before and after intervention

	IR-group-pre	IR-post group	change
	(*n* = 36)	(*n* = 36)	
Gender (M/F)	18/18	-	-
Age (year)	12.8 ± 1.8	-	-
Weight (kg)	83.79 ± 20.01	75.26 ± 17.63**	-8.53 ± 3.42
Chest C (cm)	102.15 ± 14.61	96.51 ± 9.81**	-5.63 ± 10.12
WC (cm)	101.24 ± 13.88	92.78 ± 12.46**	-8.46 ± 3.78
HC (cm)	109.66 ± 10.62*	103.18 ± 10.10**	-6.48 ± 2.70
WHR	0.92 ± 0.08**	0.96 ± 0.10**	0.04 ± 0.05
Thigh C (cm)	62.89 ± 7.84	57.18 ± 8.43**	-5.71 ± 3.18
Calf C (cm)	46.13 ± 11.24	42.18 ± 10.39**#	-3.94 ± 4.11
FM (kg)	28.36 ± 9.75	22.51 ± 8.19**	-5.85 ± 2.59
FFM (kg)	55.42 ± 11.33	52.74 ± 10.79**	-2.68 ± 1.16
SMM (kg)	50.67 ± 10.26	48.45 ± 9.84**	-2.22 ± 1.02
HOMAIS	0.27 ± 0.84	0.44 ± 0.47**	0.17 ± 0.45
HOMAβ	224.97 ± 157.31	221.10 ± 167.81**	-3.87 ± 129.08
Resting O2 (ml/min)	350.62 ± 81.85	317.15 ± 157.57	-33.47 ± 152.21
Resting CO2 (ml/min)	263.73 ± 67.75	232.95 ± 122.43	-30.77 ± 115.81

Data were reported as means ± (SD), analyzed by an independent sample t test. * *p* < 0.05, ** *p* < 0.01 pre group vs. post group

### Plasma metabolomics patterns of IR children

To further explore the effects and mechanism of lifestyle modification on obese children with IR, Q300 metabolomics was used in this study. Our metabolomics results showed that the serum profile of IR children exhibits a wide range of deviations in plasma metabolite levels.

**Fig. 3:**
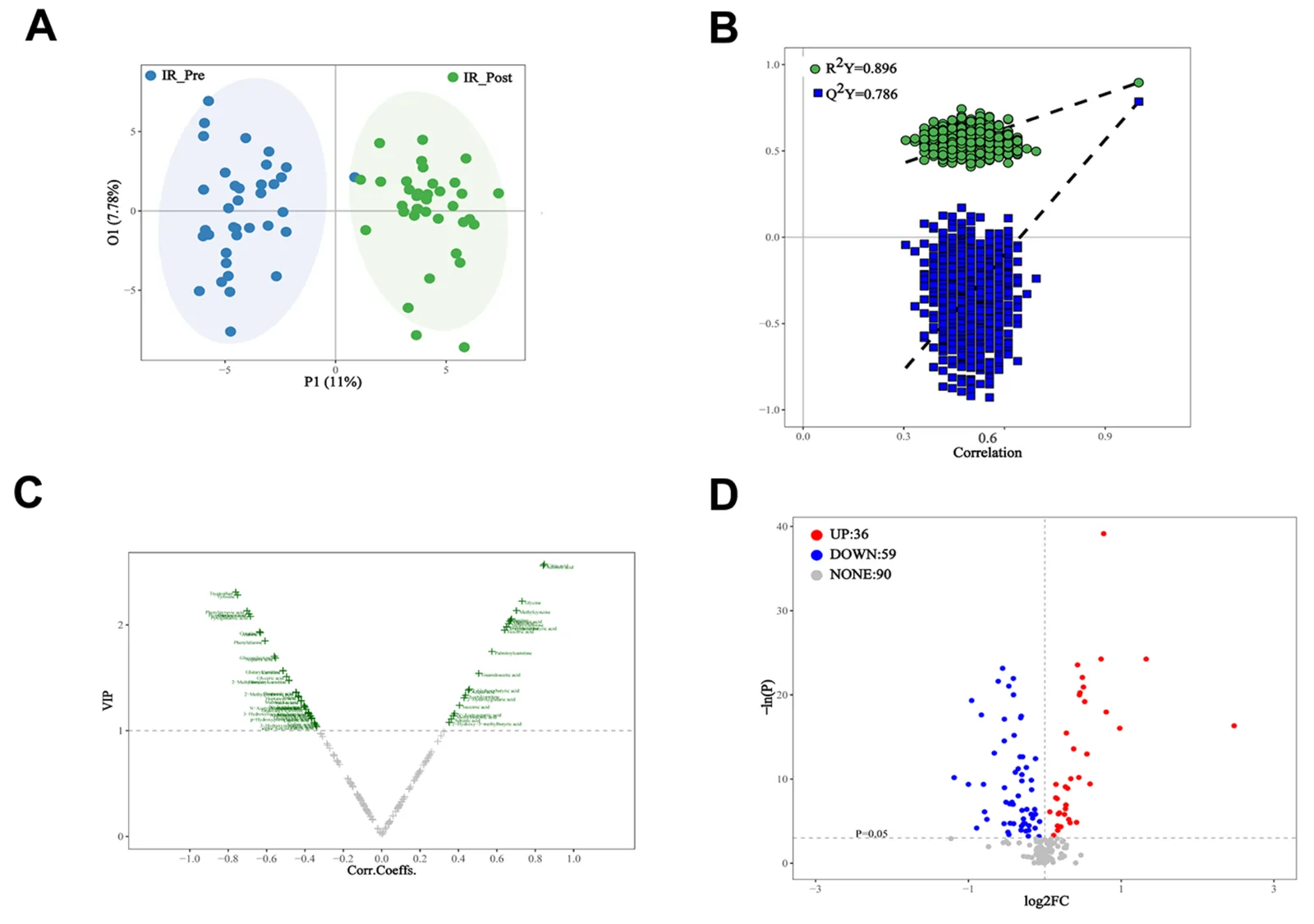
Plasma metabolome profiles of the obesity children with IR before and after weight loss intervention. (**A**) Principal component analysis (PCA) score plots; (**B**) Orthogonal projection to latent structures discriminant analysis (OPLS-DA) cross validation plot; (**C**) Volcano plots of OPLS-DA; (**D**) Volcano plots of univariate analysis

The differences between IR children before and after lifestyle modification were best described by orthogonal partial least squares discriminant analysis (OPLS-DA), with an *R2* value of 0.896 and a *Q2* value of 0.786 (Fig. 3A, B). Based on the OPLS-DA model results, a volcano plot was used to select the most important potential metabolites (Fig. 3C). Given that *VIP* > 1.0 and *p* < 0.05, 61 metabolites were identified (Supplementary Table 4). In addition, univariate statistical analysis (Student’s t test or Mann‒Whitney U test) was also used in IR children before and after lifestyle modification to identify significantly changed metabolites. We found that 36 metabolites were significantly increased and 59 metabolites were decreased in IR obese children after weight loss (Fig. 3D).

**Fig. 4:**
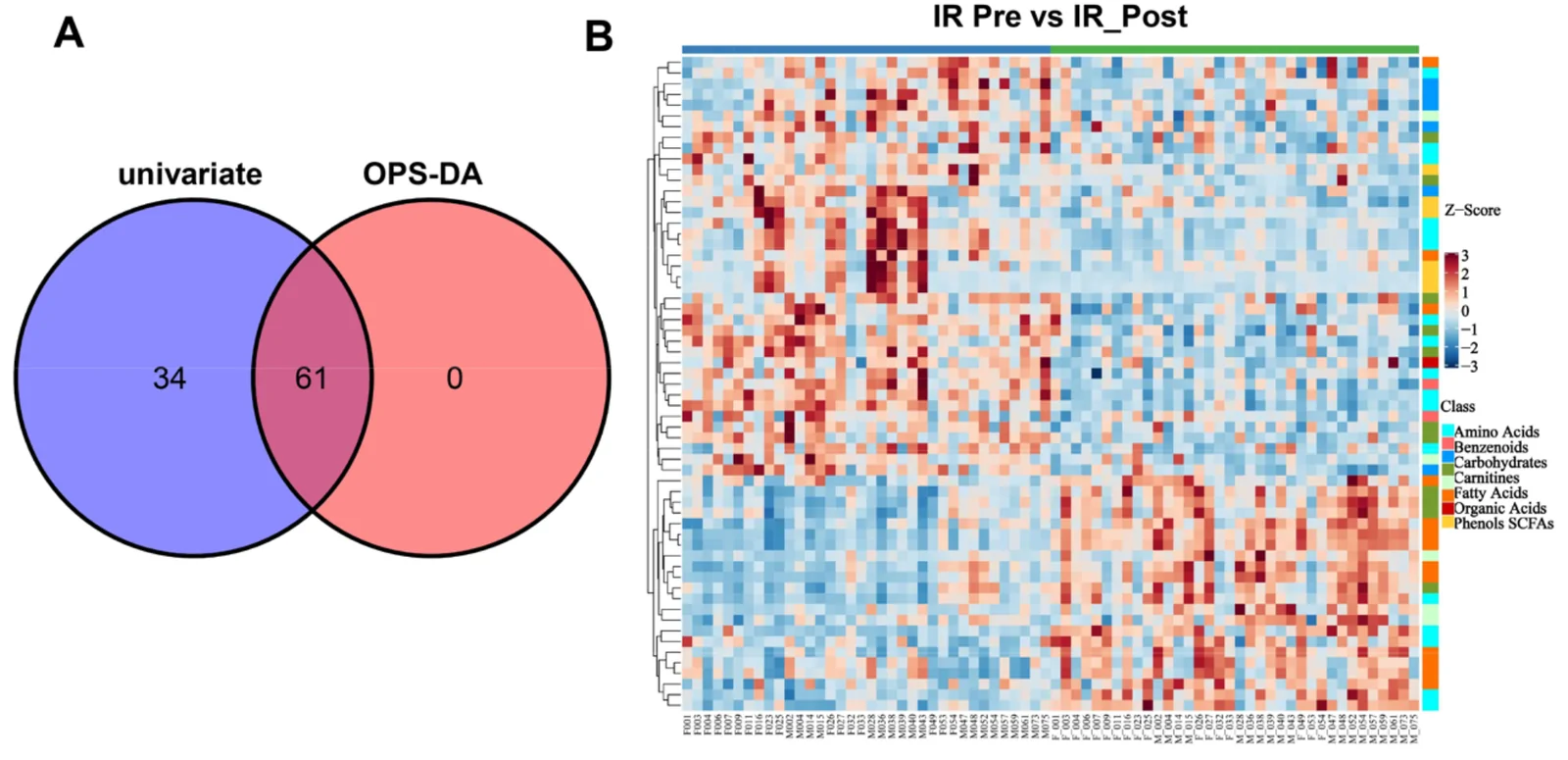
Potential plasma metabolites. (**A**) Venn plot of differential metabolites. (**B**) Z Score plot of potential biomarkers

Furthermore, to identify potential metabolites that may play a critical role in weight loss of children with IR, intersection analysis of the differential metabolites from univariate statistics and multidimensional statistics was performed. *VIP* > 1 in multidimensional statistics and *p* < 0.05 and |log2FC| ≥ 0 in univariate statistics were used as the threshold values for potential metabolites selection in this analysis. There were 61 metabolites that matched the threshold value, as shown in Fig. 4; Table 2.

**Table 2:** Potential metabolites selected

Metabolite	Class	*P*	log2FC	OPLSDA VIP
Glutamic acid	Amino Acids	0.00	-0.61	2.09
Alanine	Amino Acids	0.00	-0.40	1.91
Serine	Amino Acids	0.00	0.46	2.04
Creatine	Amino Acids	0.00	-0.96	1.92
Homocitrulline	Amino Acids	0.00	-0.53	1.21
Methylcysteine	Amino Acids	0.00	1.33	2.12
Tyrosine	Amino Acids	0.00	-0.41	2.27
Phenylalanine	Amino Acids	0.00	-0.32	1.83
Aspartic acid	Amino Acids	0.00	-0.66	1.67
Aminoadipic acid	Amino Acids	0.00	-1.19	1.14
2-Hydroxy-3-methylbutyric acid	Fatty Acids	0.00	0.98	1.06
Malic acid	Organic Acids	0.00	0.43	2.02
N-Acetyaspartic acid	Amino Acids	0.00	0.30	1.15
Azelaic acid	Fatty Acids	0.00	-0.53	1.06
alpha-Ketoisovaleric acid	Organic Acids	0.02	-0.13	1.02
Phenylpyruvic acid	Benzenoids	0.00	-0.53	2.12
Methylmalonic acid	Organic Acids	0.00	0.14	1.13
2-Hydroxyglutaric acid	Organic Acids	0.00	0.15	1.30
Glycine	Amino Acids	0.00	0.45	2.21
Guanidoacetic acid	Organic Acids	0.00	0.38	1.53
Gluconolactone	Carbohydrates	0.00	-0.31	1.69
Glyceric acid	Carbohydrates	0.00	-0.29	1.50
Pipecolic acid	Amino Acids	0.00	0.80	2.03
N-Acetylserine	Amino Acids	0.00	-0.18	1.23
N-Acetylneuraminic acid	Carbohydrates	0.00	-0.13	1.21
Valine	Amino Acids	0.00	-0.16	1.31
Pyroglutamic acid	Amino Acids	0.00	-0.55	2.06
Maltose/Lactose	Carbohydrates	0.00	-0.43	1.27
Maltotriose	Carbohydrates	0.00	-0.42	1.21
3-Hydroxybutyric acid	Organic Acids	0.00	2.48	1.97
2-Hydroxybutyric acid	Organic Acids	0.00	0.59	1.38
3-Hydroxyisovaleric acid	SCFAs	0.00	-0.12	1.05
Xylose	Carbohydrates	0.00	-0.24	1.13
Propionic acid	SCFAs	0.00	-0.35	1.35
p-Hydroxyphenylacetic acid	Phenols	0.01	-0.53	1.11
Tryptophan	Amino Acids	0.00	-0.47	2.30
Butyric acid	SCFAs	0.00	-0.24	1.21
Mandelic acid	Benzenoids	0.01	-0.26	1.15
Valeric acid	SCFAs	0.00	-0.30	1.27
Adipic acid	Fatty Acids	0.00	0.55	1.37
Aconitic acid	Organic Acids	0.00	0.77	2.54
2-Methylpentanoic acid	SCFAs	0.00	-0.51	1.34
Caproic acid	SCFAs	0.00	-0.18	1.10
Citric acid	Organic Acids	0.00	0.74	2.56
Isocitric acid	Organic Acids	0.00	0.49	1.94
Heptanoic acid	Fatty Acids	0.00	-0.46	1.30
Pyruvic acid	Organic Acids	0.01	-0.28	1.04
Oxoglutaric acid	Organic Acids	0.00	-0.17	1.12
Adrenic acid	Fatty Acids	0.00	0.34	1.10
Succinic acid	Organic Acids	0.00	0.16	1.22
Carnitine	Carnitines	0.00	-0.30	1.55
Acetylcarnitine	Carnitines	0.00	0.45	1.32
Propionylcarnitine	Carnitines	0.00	-0.83	2.09
2-Methylbutyroylcarnitine	Carnitines	0.00	-0.32	1.46
Isovalerylcarnitine	Carnitines	0.00	-0.80	1.46
3-Hydroxylisovalerylcarnitine	Carnitines	0.00	-0.12	1.16
Glutarylcarnitine	Carnitines	0.00	-0.41	1.55
Dodecanoylcarnitine	Carnitines	0.00	-0.39	1.19
Palmitoylcarnitine	Carnitines	0.00	0.28	1.73
Oleylcarnitine	Carnitines	0.00	0.51	1.97
Stearylcarnitine	Carnitines	0.00	0.52	2.00

### Pathways related to weight loss in children with IR

**Fig. 5:**
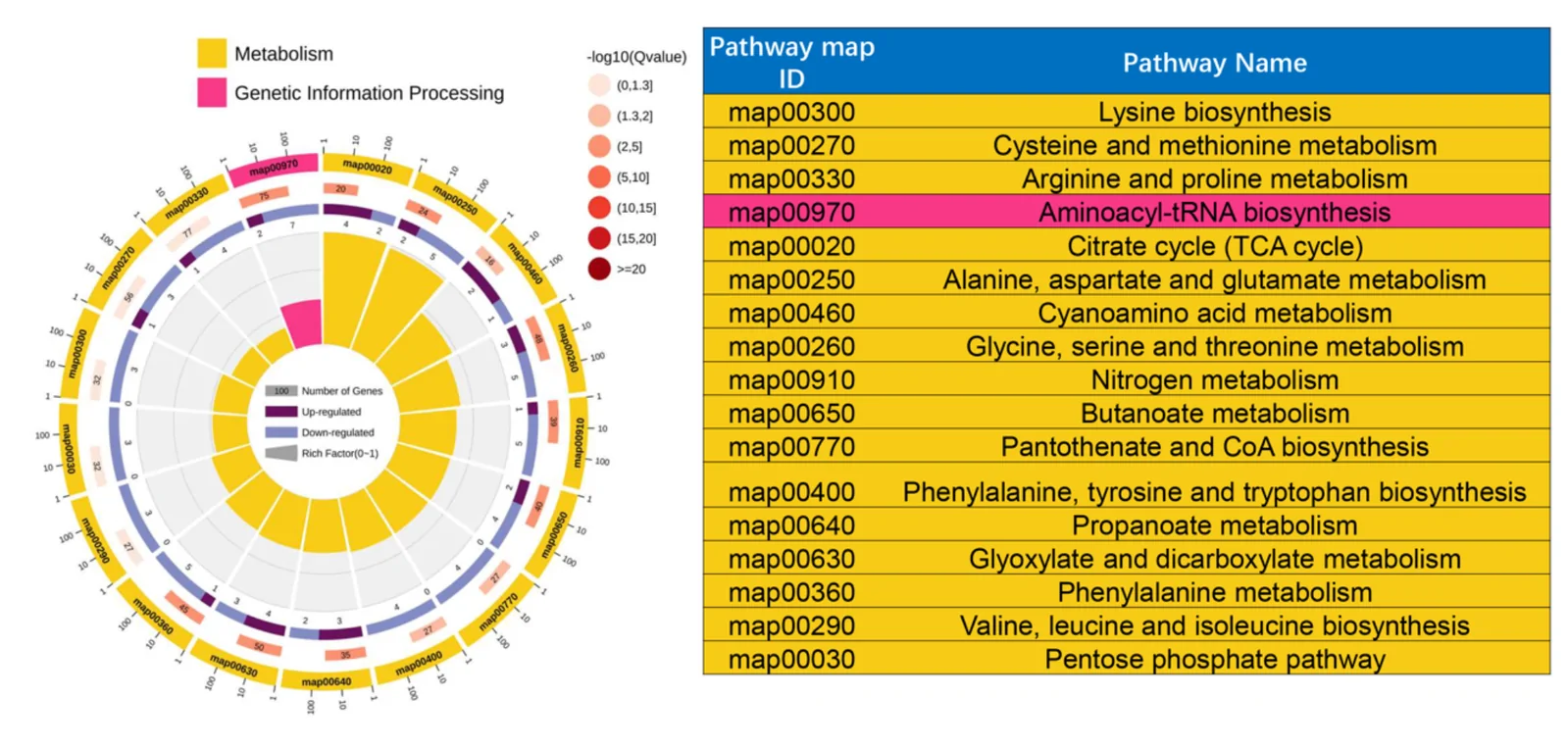
Pathway analysis plot by the HSA set in KEGG. The first lap displays significantly 17 KEGG terms (FDR < 0.1) and the numbers of the metabolites corresponds to the outer lap. The second lap displays the number of the metabolites in the genome background and P values for enrichment of the differentially expressed metabolites. The third lap displays the ratio of the upregulated metabolites (deep purple) and downregulated metabolites (light purple). The fourth lap displays the enrichment factor of each KEGG term

To understand the potential mechanism in children with IR obesity before and after weight loss, KEGG was used to analyze related metabolites. Of the 61 potential metabolites, 12 pathways (-ln(p) > 5) were significantly changed. The results of each of the 12 KEGG pathways were plotted to show the importance of the pathways (Fig. 5). The top 6 significant pathways were alanine, aspartate and glutamate metabolism; citrate cycle (TCA cycle); glycine, serine and threonine metabolism; aminoacyl-tRNA biosynthesis; and glyoxylate and dicarboxylate metabolism, which are involved in the process of IR.

### Potential biomarkers

**Fig. 6:**
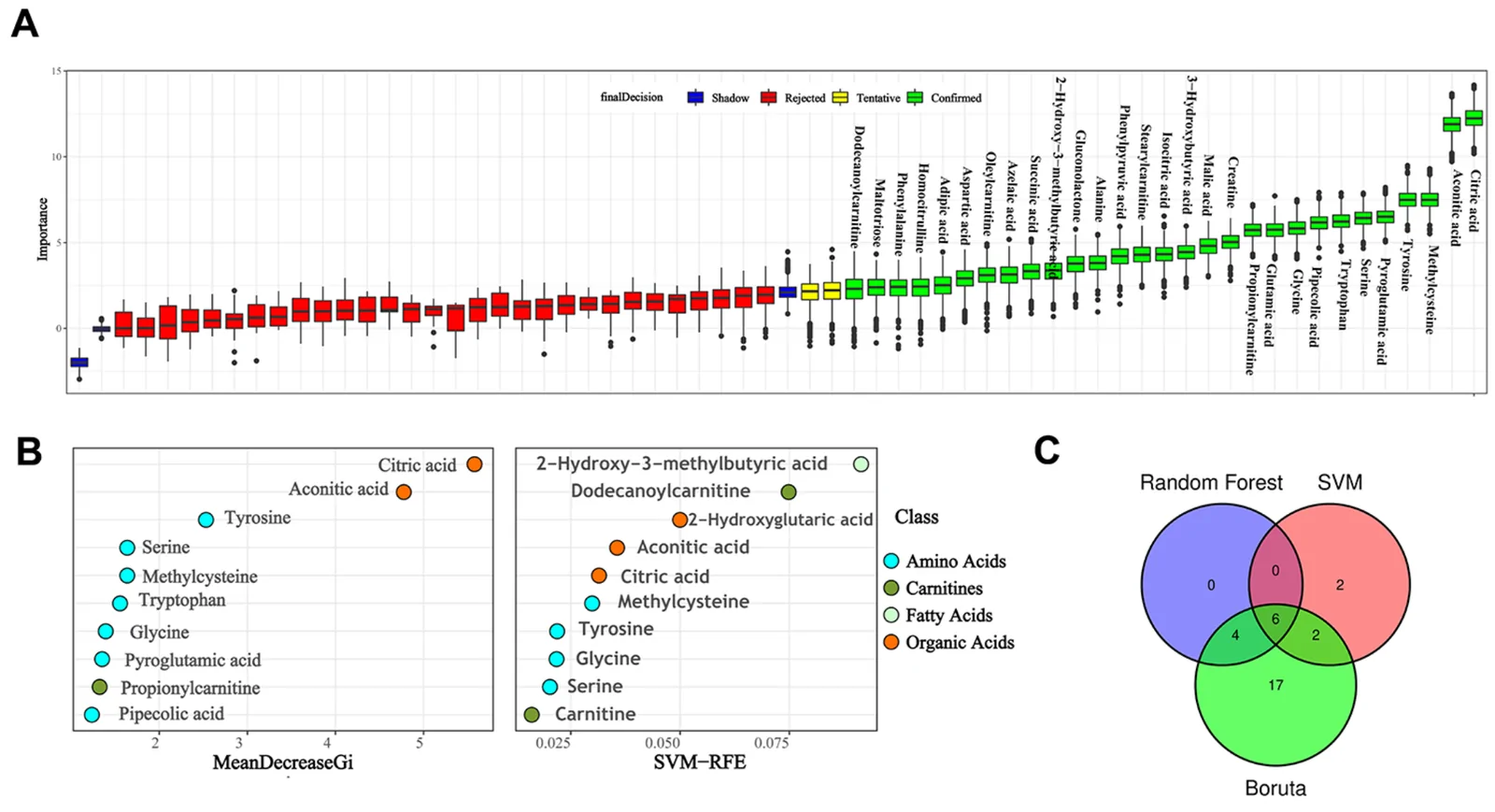
Potential marker from prediction and diagnosis model. (**A**) Data results of feature selection by Boruta analysis; (**B**) Importance scores of top 10 important differential metabolites by random forest (RF); (**C**) Importance scores of top 10 important differential metabolites by support vector machine (SVM); (D) Venn plot of differential important metabolites

**Fig. 7:**
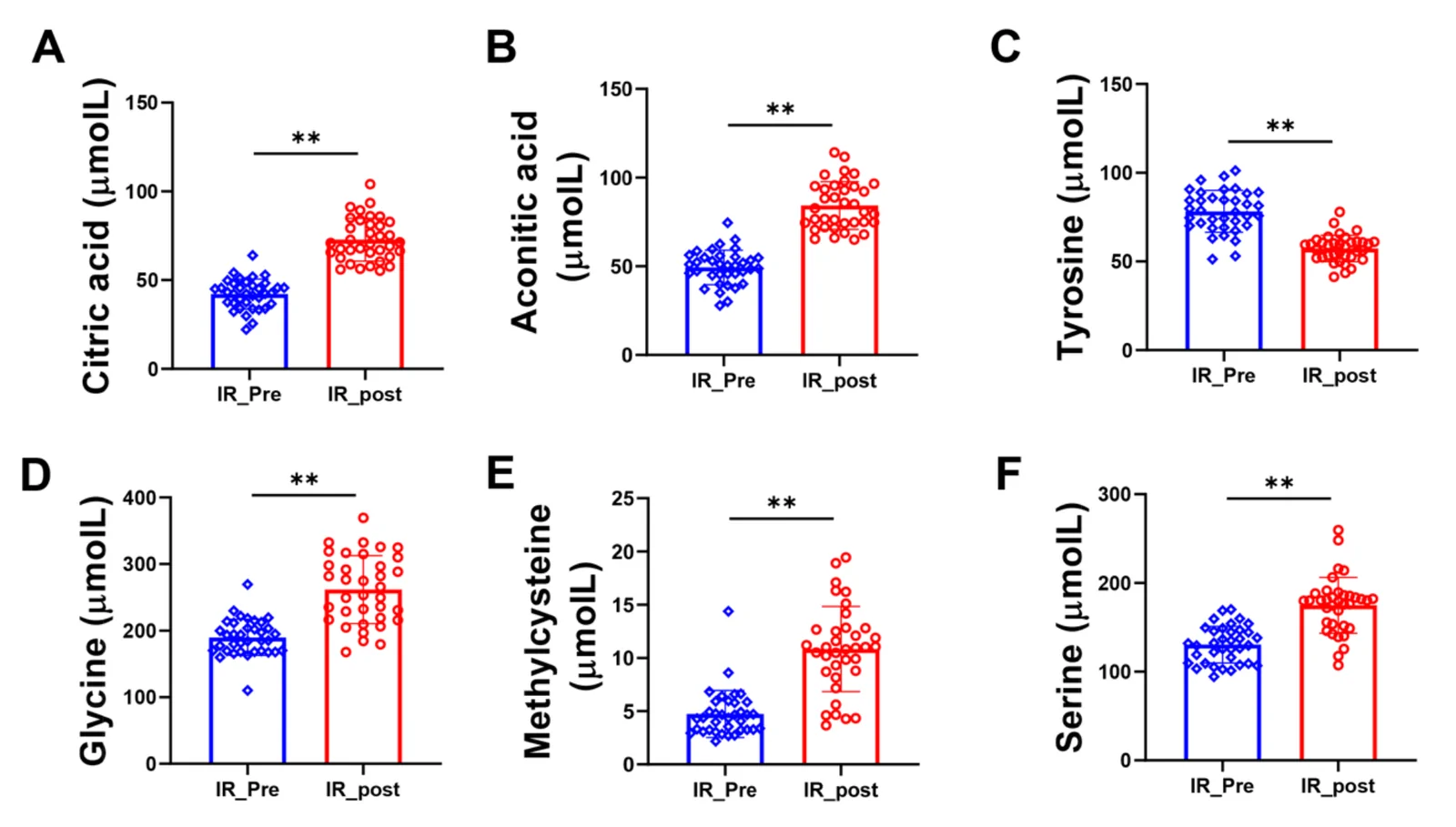
Changes in 6 most important potential biomarkers concentrations in obese children with IR following weight loss intervention. (**A**) Citric acid; (**B**) Aconitic acid; (**C**) Tyrosine; (**D**) Glycine; (**E**) Methylcysteine; (**F**) Serine. Data are presented as mean ± standard deviation. *p < 0.05 between corresponding groups. **p < 0.01 between corresponding groups

To reveal the potential biomarkers of weight loss in IR obese children, random forest (RF), support vector machine (SVM) and Boruta analyses were performed. Intersection analysis of the top 10 important metabolites of RF, SVM and Boruta analysis was performed. We obtained 6 important potential biomarkers, namely, citric acid, aconitic acid, tyrosine, serine, methylcysteine and glycine (Figs. 6 and 7).

### Associations of metabolites with major clinical parameters

**Fig. 8:**
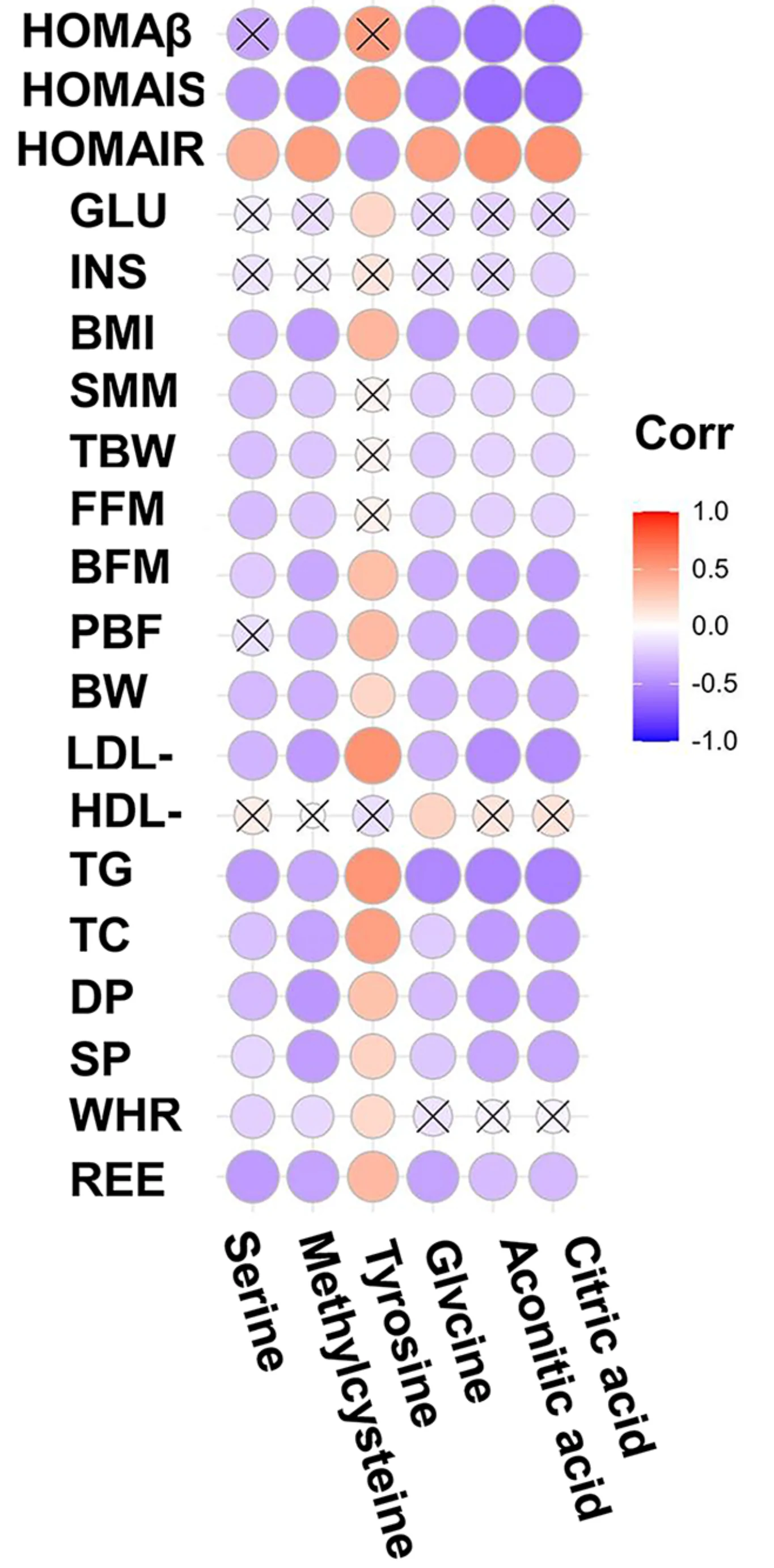
Associations of metabolites with major clinical parameters

Spearman correlation analysis revealed that metabolites were correlated with a range of clinical parameters. For example, serine, methylcysteine, glycine, aconitic acid and citric acid were mostly positively associated with HOMA-IR but inversely associated with HOMA-IS, BMI, body fat mass (BFM), BW, LDL-C, TGs, TC, diastolic pressure (DP), systolic pressure (SP) and REE. Tyrosine was mainly negatively associated with HOMA-IR and positively associated with HOMA-IS, BMI, BFM, fat percentage (PBF), BW, LDL-C, TGs, TC, DP, SP, WHR and REE (Fig. 8).

## Discussion

In the current study, we outlined the metabolite features of obese IR children after weight loss. Moreover, we further demonstrated that serine, methylcysteine, glycine, aconitic acid, tyrosine and citric acid were the most significantly changed metabolites in the process of weight loss. Additionally, we demonstrated the association between 6 important metabolites and major clinical parameters.

Numerous studies have suggested that lifestyle modification is associated with variable effects on weight loss and body health (Wadden et al. 2020)^,^ (Smith et al. 2020) (Kushner and Ryan 2014) (Kushner 2018). In a previous study, we found that 4 weeks of weight loss intervention could reduce body weight and contributed to cardiometabolic health in children with metabolic syndrome (Liu et al. 2021). Consistent with these findings, in this study, we found that 4 weeks of weight loss intervention could effectively decrease body weight, BMI, HOMA-IR and body circumference in obese IR children (Fig. 2). These findings suggest that weight loss intervention is an effective method for weight loss in obese children, including those with IR and metabolic syndrome.

The significant alteration of the metabolome that occurs in obesity is related to wellbeing, and profiling can be used to identify clinically significant heterogeneity (Cirulli et al. 2019). Consistent with this viewpoint, our metabolome data showed that there was profound change of the metabolome in IR children before and after lifestyle modification (Fig. 3). In addition, univariate statistics and multidimensional statistics were used to obtain 61 important potential metabolites (Fig. 4). Based on these metabolites, 12 related pathways were enriched in children with IR obesity before and after weight loss invention (Fig. 5). Alanine, aspartate and glutamate metabolism plays an important role in the pathogenesis of metabolic syndrome (Sookoian and Pirola 2012). Consistent with this finding, metabolism of alanine, aspartate, and glutamate was also significantly changed in obesity children with IR following the intervention (Fig. 5). The TCA cycle is the foremost critical metabolic pathway that supplies energy to the body and is the ultimate common oxidative pathway for carbohydrates, fats and amino acids. The TCA cycle pathway has been associated with obesity, prediabetes and IR (Zhang et al. 2021) (Adams et al. 2009) (Muoio and Newgard 2008). Guasch et al.(Guasch-Ferré et al. 2020) found that TCA cycle-related metabolites (such as aconitate, citrate, isocitrate and malate) were significantly connected with type 2 diabetes risk in a population consuming the mediterranean diet. In line with these findings, our results revealed that the TCA cycle pathway was significantly changed in children with IR obesity before and after weight loss intervention (Fig. 5). Glycine metabolism is an important pathway involved in weight loss. Glycine concentrations in serum was increased after which loss(Adeva-Andany et al. 2018). As expected, our pathways analyses confirmed this concept. In addition, we found that glyoxylate and dicarboxylate metabolism and aminoacyl-tRNA biosynthesis were all involved in the weight loss process of children with IR obesity after lifestyle modification (Fig. 5). OuYang et al. (OuYang et al. 2020) found that high glucose intake altered glyoxylate and dicarboxylate metabolism in a prediabetes rat model. Lind et al. (Lind et al. 2022) found that aminoacyl-tRNA biosynthesis was related to insulin sensitivity and was a pathway that was highlighted between subjects with normal weight and obesity. These interesting results all support our findings that the above metabolic pathways are involved in the weight loss process of children with IR obesity.

To obtain the most important metabolites, RF, SVM and Boruta analyses were performed. Citric acid, aconitic acid, tyrosine, serine, methylcysteine and glycine were obtained in this analysis. Citric acid is an intermediate in the citric acid cycle, and due to its antioxidant properties, it is widely used as an excipient in pharmaceutical preparations. Muroyama et al. (Muroyama et al. 2003) found that a 12-week intervention with daily intake of thiamin, arginine, caffeine and citric acid significantly decreased serum TGs, abdominal visceral fat and percent body fat in healthy subjects with a high percentage body fat (> 25.0%). Zou et al. (Zou et al. 2020) found that the concentrations of citrate, aconitic acid and α-ketoglutarate were lower in skeletal muscle from severely obese women with type 2 diabetes than in lean nondiabetic subjects’ skeletal muscle. Similar with these results, we found that the concentrations of citrate and aconitic acid were significantly decreased in obese IR children after the weight loss program (Fig. 6). These results suggested that the lifestyle modification had good effects.

Serine is a nonessential amino acid but plays an important role in the metabolic processes that burn glucose and fatty acids for energy (Gao et al. 2018). Obese participants showed lower serine levels than those of lean controls (Fridman et al. 2021). Aging mice treated with serine had significantly decreased concentrations of IL-6, IL-1β and leptin in the serum compared with control mice (Zhou et al. 2018). In addition, serine is essential for the production of glycine (He et al. 2023), which is an important amino acid in cardiometabolic diseases. A deficiency in glycine intensifies the progression of obesity, hypercholesterolemia, hyperglycemia, and atherosclerosis. Conversely, glycine supplementation significantly ameliorates glycemic regulation, dyslipidemia, cardiovascular function, and hepatic steatosis (Rom et al. 2018). Plasma glycine levels are lower in IR individuals than in healthy individuals. However, after exercise, weight loss and metformin treatment, IR was improved, and the glycine concentration was increased (Adeva-Andany et al. 2018). Consistent with these results, we found that the concentrations of serine and glycine were significantly increased after weight loss intervention in obese children with IR (Fig. 7).

Methylcysteine is a bioactive substance found in garlic and human blood. Castro et al. found that diabetic rats treated with methylcysteine had significantly decreased blood glucose and NF-ΚB levels (Castro et al. 2021). In this study, we found that methylcysteine was significantly increased and was associated with HOMA-IR and HOMA-IS in children with IR obesity after weight loss intervention (Figs. 7 and 8). These findings are consistent with a previous report that the levels of metabolites related to S-methylcysteine were significantly increased after weight loss intervention in children and adolescents with severe obesity (Sohn et al. 2022).

Tyrosine is another important metabolite that is postive associated with HOMA before and after one year of lifestyle changes in obese children (Kretowski et al. 2016) (Hellmuth et al. 2016). Furthermore, Mohorko et al. (Mohorko et al. 2015) found that tyrosine had significant strong associations with BMI and waist circumference and was significantly higher in young adults with metabolic syndrome than in healthy young adults. Consistent with these reports, our results showed that tyrosine was significantly decreased and was associated with BMI and HOMA-IS in obese children with IR after weight loss intervention (Figs. 7 and 8).

While the present study provides valuable insights into the metabolomic responses to weight loss interventions in obese children with insulin resistance, several limitations must be considered when interpreting our findings. Firstly, our sample size, though adequate for the metabolomic analysis, may limit the generalizability of the results to broader populations. The relatively short duration of the intervention may not capture the long-term sustainability of weight loss or the stability of the metabolic changes observed. Additionally, the study’s design did not allow for differentiation between the effects of diet, exercise, or their combination on the metabolomic profiles. The potential influence of genetic predispositions and environmental factors on individual responses to the intervention was not explored, which could be crucial given the known heterogeneity in responses to weight loss strategies. Furthermore, the lack of a control group that did not receive the intervention limits our ability to attribute the observed changes solely to the weight loss program. Lastly, while we employed rigorous analytical methods, the complexity of metabolomic data and the multiple comparisons made increase the likelihood of type I errors. Future studies with larger cohorts, longer follow-up periods, and inclusion of genetic and environmental covariates could help overcome these limitations and provide a more comprehensive understanding of the metabolic changes associated with weight loss in obese children with insulin resistance.

## Conclusion

Our findings indicate that there were significantly different metabolomic profiles in obese children with IR after weight loss intervention, providing insights into the clinical parameters and metabolite mechanisms involved in weight loss interventions.

## Electronic supplementary material

Below is the link to the electronic supplementary material.

Supplementary Material 1

Supplementary Material 2

Supplementary Material 3

Supplementary Material 4

Supplementary Material 5

## Data Availability

No datasets were generated or analysed during the current study.
